# The International Union of Pure and Applied Chemistry and its role on the world stage

**DOI:** 10.1093/nsr/nwab036

**Published:** 2021-03-05

**Authors:** Christopher Brett

**Affiliations:** Professor, Department of Chemistry, University of Coimbra, Portugal; IUPAC President

It is a pleasure to write this contribution for *National Science Review* and to share information about the different pathways being followed by the International Union of Pure and Applied Chemistry (IUPAC) toward a worldwide sustainable society.

IUPAC was founded in 1919 with a strong impetus from chemical industries that recognized the importance of using a common language, particularly for chemical compounds, so that they would be able to communicate with each other. After IUPAC’s beginnings in Europe, it expanded and has become a truly worldwide organization, representing the global interests of the chemistry community and scientific communities associated with chemistry. These include academic institutions, industry and society at large. IUPAC’s vision of being a global indispensable source of chemistry is embodied in our mission to provide objective scientific expertise and develop the essential tools for applying and communicating chemical knowledge to benefit humankind and the world. This is achieved through our providing a common language for chemistry, advocating the free exchange of scientific information, and fostering sustainable development. Scientific excellence and objectivity, diversity and inclusiveness, are IUPAC core values that are particularly important in order to give objective advice and recommendations as a response to the many challenges that are faced by chemists, the scientific community and society.

Our centenary year, 2019, was also the International Year of the Periodic Table of the Chemical Elements (IYPT), of which IUPAC was the leading partner. The many events held all over the world demonstrated the enthusiasm for chemistry by all, of different ages and from all parts of society. It enabled us to show the fascination for the chemical elements and emphasized how many positive things they do for us in contributing to our lives. A Periodic Table Challenge was held, taken by over 65 000 people in 136 countries. Following its huge success, a second edition was launched in June 2020 and has been equally successful; there are now versions in Chinese, Arabic, Spanish and Russian. The Chinese version has already been taken by several thousand people.

Other centenary year initiatives that continued in 2020 and are continuing in 2021 are the Global Women's Breakfast (GWB), and the Top Ten Emerging Technologies series of articles. Highlighting these new technologies is a way for IUPAC to help show how chemists are contributing to adapting the chemical industry to new needs, with new materials and using processes that are less energy intensive. The Periodic Table of Younger Chemists, another IYPT initiative, in collaboration with the International Younger Chemists’ Network, has led directly to a new project: ChemVoices, the international voice of the younger chemists, which began a series of webinars in September 2020.

Core IUPAC activities continue, of course, and we continue to develop chemical nomenclature, with recommendations and technical reports, all open access, appearing in our journal *Pure and Applied Chemistry.* The ‘Color Books’, provide standards and standardized nomenclature in different areas of chemistry. It will be particularly important to develop Cheminformatics standards, of which the international chemical identifier (InChI) is an excellent example. *Pure and Applied Chemistry* also publishes themed scientific issues, many of them linked to important contributions made in IUPAC-endorsed conferences. Our publication *Chemistry International* highlights important news items and the results of our projects, and *Chemistry Teacher International* is our publication for educators.

We value our collaborations with international organizations such as UNESCO, International Science Council (ISC), International Bureau of Weights and Measures (BIPM), the Organization for the Prohibition of Chemical Weapons (OPCW) and the International Union of Pure and Applied Physics (IUPAP). For example, IUPAP collaborates with us to determine when a new element has been synthesized and identified. IUPAC was awarded the Hague award of the OPCW in 2019 for its work in collaboration with OPCW to further the peaceful uses of chemistry.

IUPAC’s 2300 volunteers are from all over the world, representing scientists in different areas of chemistry and multidisciplinary areas with the best skills and knowledge. We have 54 national adhering organizations (NAOs), and over 800 Affiliate Members, Company Associates and Associated Organizations. Together we identify current and emerging scientific issues and problems and reach consensus on approaching and solving them. The Chinese Chemical Society joined IUPAC as an NAO in 1979. Since then, chemical sciences and technology in China have undergone fast development and now China is one of the most important countries in the chemistry field worldwide. China has played an important role in IUPAC. Prof. Chunli Bai has served in the IUPAC bureau and executive committee. Prof. Qifeng Zhou has served as IUPAC President in 2018–2019; under his leadership, IUPAC coordinated the IYPT activities with great success.

The world is facing many challenges in health, food and the environment. The need for a multidisciplinary and global approach, in all senses, becomes paramount. Particular challenges at the moment in chemistry are sustainability, new materials and reduction in energy needs, whilst moving to renewable energy, which need to integrate new technologies. We are fomenting activities in green chemistry and sustainable development, building on our links with industry. We have projects in responsible care, education and training. We have a particular focus on inspiring younger chemists and reaching out to those parts of the world in greatest need, a principal tenet of a sustainable society. Within chemistry, sustainable chemistry must be our guide, not only in the context of a circular economy but also by first identifying what we need and then examining which ways to achieve it, tailoring the processes and cycles to demand rather than to supply, making them more efficient and sustainable.

We can only achieve these goals, which are crucial for a sustainable society, through the strong collaboration of all who are linked to the chemical sciences. Please visit our website www.iupac.org for more information about our activities in IUPAC and internationally. We very much look forward to increased involvement by Chinese chemists in our projects for the future.

